# Investigating the Mullins Effect and Energy Dissipation in Magnetorheological Polyurethane Elastomers

**DOI:** 10.3390/ijms21155318

**Published:** 2020-07-27

**Authors:** Alex Elías-Zúñiga, Luis M. Palacios-Pineda, Imperio A. Perales-Martínez, Oscar Martínez-Romero, Daniel Olvera-Trejo, Isaac H. Jiménez-Cedeño

**Affiliations:** 1 Mechanical Engineering and Advanced Materials Department. School of Engineering and Science, Tecnologico de Monterrey, Ave. Eugenio Garza Sada 2501, Monterrey C.P. 64849, N.L., Mexico; anel.perales@tec.mx (I.A.P.-M.); oscar.martinez@tec.mx (O.M.-R.); daniel.olvera.trejo@tec.mx (D.O.-T.); 2 División de Estudios de Posgrado e Investigación, Tecnológico Nacional de México/Instituto Tecnológico de Pachuca, Carr. México-Pachuca Km 87.5, Pachuca, Hidalgo C.P. 42080, Mexico; luis.pp@pachuca.tecnm.mx; 3 Engineering Department, Safran Mexico, Calle Ishikawa #1201 Parque Ind. Supra Chihuahua, Chihuahua C.P. 31183, Mexico; isaac-h.jimenez-c@safrangroup.com

**Keywords:** pseudo-elastic material model, damage function, energy dissipation, mullins effect, magnetorheological elastomer

## Abstract

The aim of this article was to investigate the mechanical performance of magnetorheological polyurethane elastomers reinforced with different concentrations of carbonyl iron microparticles (CIPs) in which stress softening, energy dissipation, residual strains, microparticles orientation, and magnetic flux density effects will be considered. Other aspects, such as the determination of the dissipated energy during cyclic loading and unloading, were investigated by considering a pseudo-elastic network model that takes into account residual strains, magnetic field intensity, and the isotropic and anisotropic material behavior. Theoretical predictions confirmed that the material shear modulus becomes sensitive not only for higher concentrations of CIPs added into the elastomer material matrix, but also to the magnetic flux intensity that induces attractive forces between CIPs and to the strong bonds between these and the elastomer matrix. It was also found that the addition of CIPs when embedded into the polymer matrix with a predefined orientation enhances the material shear modulus as well as its capacity to dissipate energy when subjected to magnetic flux density in loading and unloading directions.

## 1. Introduction

The aim of this paper focused on investigating the Mullins effect, residual strains, and energy dissipation in uniaxial loading–unloading cycles by considering the concentration of carbonyl iron particles (CIPs) added into an elastomer matrix, their orientation influence, and the impact of applying a magnetic flux density in the loading direction. In this sense, Coquelle et al. investigated the Mullins effect in Rhodorsil RTV 1062S elastomer reinforced with 10% volume of carbonyl iron particles subjected to cyclic uniaxial extension tests without the application of a magnetic flux density [1]. They found that the Mullins effect, after a few loading–unloading cycles of anisotropic samples, is close to that of the isotropic ones and that the usage of a coupling agent strongly changes the slope of the first traction. Then, Coquelle and Bossis [2] performed cyclic uniaxial extension tests to study and model the mechanism that promotes the detachment of the polymer matrix from the CIPs. They found that after the first loading and unloading cycle, new adhesion of the elastomer matrix with the CIPs surface occurs. They also noticed that the composite material during the second loading and unloading cycle is different from the one exhibited in the first cycle since some elastomer-to-particle bonds were broken. They related this material molecular damage to the Mullins effect and performed some computer simulations via the finite element method (FEM) to model the stress-softened behavior experienced by the composite material. They also found that the quality of the bonds between elastomer and CIPs does not influence the material modulus value promoted by magnetic flux density. Considering dipole–dipole interaction of the CIPs, Melenev et al. [3] proposed a phenomenological rheological model that takes into account linear elastic effects and internal dry friction and makes both material networks deform affinely. In order to evaluate the accuracy of their proposed rheological model, they performed uniaxial cyclic tests on silicone rubber elastomer samples reinforced with a 30 vol% of CIPs (2 to 5 µm). The material samples were cured without the action of a magnetic field in order to obtain a homogeneous distribution of isotropic CIPs. To capture stress softening and residual strain effects, they used a three branch macrorheological model that requires the determination of eleven material constants to predict experimental data. Based on their model predictions, they concluded that the deviation between experimental tests and theoretical predictions is attributed to the material viscolelastic effects that were not included in the material model. In this sense, Shariff and Bustamante [4] also proposed an anisotropic phenomenological model, which is based on direction-dependent damage parameters that depend on the strain history to simulate the Mullins effect for magnetoactive elastomers. Combining small and wide-angle X-ray scattering techniques (SAXS and WAXS), Jiang et al. [5] performed in situ experimental measurements of an isotropic copolymer sample BA6500-Fe_3_O_4_4.9 subjected to a uniaxial loading–unloading cycle. They found that during the loading process, the copolymer grafted nanoparticles (magnetite, Fe_3_O_4_) were forced to be highly oriented along the tensile direction, revealing strain-hardening behavior because the applied stress is transferred from the copolymer matrix to the oriented Fe_3_O_4_ nanoparticles. They also found that during unloading, the orientation degree of the Fe_3_O_4_ nanoparticles and the stress magnitude decrease to recover the material entropy. Later, Xu et al. [6] confirmed that when a magnetosensitive polymer gel is subjected to cyclic shear loading, the transition from stress-softened to stress hardening is mainly due to the applied magnetic field. In fact, they found that the stress hardening effect appears at the unloading stage with or without the application of an external magnetic field, mainly due to the residual strain elasticity.

By performing compression and bending tests in magnetorheological material samples developed by considering commercial polydimethylsiloxane (PDMS) elastomer reinforced with CIPs (average size of 45 µm) concentrations of 20, 40, 60, and 80 wt%, Bellelli and Spaggiari [7] found that the anisotropic samples when subjected to compression tests exhibit higher stiffness than do isotropic ones. Recently, an MRE material was developed by mixing CIPs and flax fibers with a PDMS elastomer matrix material with an enhancement of the rheological and mechanical properties when subjected to an external magnetic field [8].

Although previous works have considered cyclic loading, they have not compared stress-softening and residual strain effects of magnetorheological elastomers reinforced with CIPs homogeneously dispersed with respect to the impact that microparticles alignment has on the material response. Here, cyclic uniaxial loading–unloading tests were performed in isotropic and anisotropic magnetorheological polyurethane (PU) elastomer samples reinforced with CIPs concentrations of 20, 35, 50, 65, and 80wt% in order to investigate stress-softening, residual strain, and energy dissipation, with and without the application of a magnetic flux density. Some modifications to the phenomenological pseudo-elastic network model for the Mullins effects derived in [9] were adapted to consider residual strains, magnetic field intensity, and material isotropic and anisotropic behavior. Furthermore, the energy dissipation factor *E* is defined in order to quantify the differences among the tested material samples for the various concentrations of CIPs in order to investigate if there exists debonding between the CIPs and the polymer matrix, since a decrease in the material shear modulus is evident after the application of the first loading and unloading cycle.

## 2. Finite Elasticity

### Basic Definitions

An isochoric deformation of an incompressible elastic material is described by

(1) $$x_{i}=\lambda_{i}X_{i},\;i=1,2,3$$

in which **X** =*X*_i_**e**_i_ defines the undeformed reference configuration of a body in a rectangular Cartesian frame *ϕ* = {*O*; **e**_i_} with origin *O* and orthonormal basis **e**_i_, **x** =*x*_i_**e**_i_ denotes the current configuration of the body when subjected to a prescribed deformation, and *λ*_i_ are the principal stretches in *ϕ*. The Cauchy–Green deformation tensor **B** = **FF^T^** is defined as

(2) $$B=\lambda_{1}^{2}e_{11}+\lambda_{2}^{2}e_{22}+\lambda_{3}^{2}e_{33},$$

where $e_{jk}\equiv e_{j}⊗e_{k},\;e_{i}$ are the orthonormal principal directions, and **F** represents the deformation gradient tensor. It is well known that in the undeformed state **F** = **1** for all isochoric deformations [10]. The principal invariants *I*_k_ of **B** are defined by

(3) $$I_{1}=trB,\;I_{2}=\frac{1}{2}(I_{1}^{2}-tr(B^{2})),\;I_{3}=\det B,$$

where *tr* denotes the trace operation, and $I_{3}\equiv 1$ for all deformations of an incompressible material.

## 3. Stress-Softening Model

To characterize the virgin material response of an incompressible and hyperelastic material under the action of an applied magnetic flux density, the following constitutive equation is considered [11]

(4) $$T_{0}=-pI+\lambda_{i}\frac{\partial W^{elastic}}{\partial \lambda_{i}}-\frac{\lambda_{i}}{2\mu }\frac{\partial F}{\partial \lambda_{i}}B^{Applied}•B^{Applied},$$

where **T_0_** is the Cauchy stress, *p* is an undetermined pressure, *λ*_i_ represents the principal stretches in the material domain, F is the deformation gradient, B^Applied^ is the magnetic flux density, *µ* defines the material magnetic permeability defined as $\mu =4\pi {\left(10\right)}^{-7}\mu_{r},$ where *µ*_r_ represents the material relative permeability constant, and *W*^elastic^ denotes the isotropized Helmholtz free energy density for a reinforced material, given as [12,13]

(5) $$W^{elastic}=(1-f)W_{iso}(I_{1})+f\left(\frac{A_{1}}{3}(I_{1}-3)+\frac{A_{2}}{9}{\left(I_{1}-3\right)}^{2}-\frac{2A_{1}}{3}\ln\sqrt{I_{3}}\right),$$

where *f* is the volumetric fraction, *A*_1_ and *A*_2_ are the isotropized material constants, and *W*_iso_(*I*_i_) is an expression that provides the virgin material isotropic strain energy density. When the material is subjected to a flux magnetic field, the magnetic energy density is expressed as

(6) $$W^{magnetic}=-\frac{1}{2\mu }FB^{Applied}•B^{Applied}.$$

Therefore, the total energy to which the material is subjected can be determined by adding Equations (5) and (6), this gives

(7) $$W_{T}=(1-f)W_{iso}(I_{1})+f\left(\frac{A_{1}}{3}(I_{1}-3)+\frac{A_{2}}{9}{\left(I_{1}-3\right)}^{2}-\frac{2A_{1}}{3}\ln\sqrt{I_{3}}\right)-\frac{1}{2\mu }FB^{Applied}•B^{Applied}$$

That is the same expression introduced in [10]. Of course, the term *W*_iso_(*I*_i_) of Equation (7) can adopt several forms depending on the material model under consideration. For instance, for the case of a neo-Hookean material model, the strain-energy density is given as:

(8) $$W_{iso}=\frac{\mu_{0}}{2}(I_{1}-3)+c_{1},$$

where *µ*_0_ is the virgin material shear modulus. Another material model that is commonly used for its accuracy in predicting the hyperelastic behavior of elastomeric materials is the well-known amended non-Gaussian strain energy density model [14], given by:

(9) $$W_{iso}(I_{1})=\mu_{0}\left[N_{8}\left(\beta \lambda_{r}+\ln\left(\frac{\beta }{\sinh\beta }\right)\right)-\ln\left(\frac{\beta }{\lambda_{r}}\right)\right]+c_{1}.$$

Here, *c*_1_ represents an energy constant, *N*_8_ is the material chain number of links, and *β* is the inverse of the Langevin function L(*β*) described by:

(10) $$\lambda_{r}=L(\beta )\equiv \cot\beta -\frac{1}{\beta }$$

with the relative chain-stretch $\lambda_{r}$ defined as:

(11) $$\lambda_{r}=\frac{\lambda_{chain}}{\sqrt{N_{8}}},\;\mathrm{with}\;\lambda_{chain}=\sqrt{\frac{I_{1}}{3}}.$$

To account for the material damage at which the material is subjected when external loads and magnetic energy density fields are applied, the pseudo-elastic model proposed by Ogden–Roxburgh in [15] is used. This model is based on the assumption that the total energy density function $W_{T}$ is related to the pseudo-total energy function ${\bar{W}}_{T}$ by the expression:

(12) $${\bar{W}}_{T}(\lambda_{1},\lambda_{2},B_{i},\eta )=\eta W_{T}((\lambda_{1},\lambda_{2},B_{i})+\phi (\eta )+\eta_{1}\hat{W}((\lambda_{1},\lambda_{2},\phi_{D}(\xi_{a})),$$

where *B*_i_ is the magnetic flux density aligned along the principal i-direction, and $W_{T}(\lambda_{1},\lambda_{2},B_{i})$ is the primary loading path energy density function for which the softening variable *η* and the residual strain variable *η*₁ are inactive. Both variables have the value of one on the loading path and depend on the maximum previous strain value, ϕ(η) and $\phi_{D}(\xi_{a})$ represent smooth damage functions that vanish on the virgin path, i.e.,ϕ(1)=0, and $\phi_{D}(0)=0,$ and $\hat{W}(\lambda_{1},\lambda_{2},\phi_{D}(\xi_{a}))$ is the residual strain energy density that depends on a damage function related to material residual strain effects [16]. Next, we recall from pseudo-elasticity theory the relationship

(13) $$-\phi^{\prime }(\eta )=W_{T}((\lambda_{1},\lambda_{2},B_{i}),$$

introduced in [15] and consider the damage function model:

(14) $$-\phi^{\prime }(\eta )=-{\left(\frac{1}{b}\ln\eta \right)}^{2}+W_{T\max},$$

proposed in [9] that provides the damage variable *η*:

(15) $$\eta =e^{-b\sqrt{W_{T\max}-W_{T}}},$$

where *b* is a positive material softening parameter, and $W_{T\max}$ is the maximum energy density value at the point for which the material is unloaded from the loading path. Therefore, the damage function ϕ(η) can be determined as a function of the material energy density given by Equation (14). This yields:

(16) $$\phi (\eta )=\frac{e^{-b\sqrt{W_{T\max}-W}}}{b^{2}}\left(2-b^{2}W_{T}+2b\sqrt{W_{T\max}-W}+e^{b\sqrt{W_{T\max}-W}}(b^{2}W_{T\max}-2)\right).$$

To find the residual strain energy density $\hat{W}(\lambda_{1},\lambda_{2}\phi_{D}(\xi_{a})),$ we assume that the residual strain energy density expression that accounts for the damage material mechanism is of the form:

(17) $$\hat{W}(\lambda_{1},\lambda_{2},\phi_{D}(\xi_{a}))=\sum_{a=1}^{3}\left(\xi_{a}(\Delta_{a}^{n}-\lambda_{a}^{n})+\phi_{D}(\xi_{a})\right),$$

where $\phi_{D}(\xi_{a})$ represents a damage function related to residual strain effects $\lambda_{res},\;\Delta_{a},\;a=1,2,3,$ are the maximum values of the principal stretches at which unloading begins on the primary loading path, and *n* is a positive scaling constant. By using the Ogden–Roxburgh [15] pseudo-elastic theory, we require that

(18) $$\frac{\partial \hat{W}}{\partial \xi_{a}}=0,$$

thus, we have:

(19) $$\frac{\partial \hat{W}}{\partial \xi_{a}}=(\Delta_{a}^{n}-\lambda_{a}^{n})+\frac{\partial \phi_{D}(\xi_{a})}{\partial \xi_{a}}=0.$$

Solving Equation (19), yields

(20) $$-\frac{\partial \phi_{D}(\xi_{a})}{\partial \xi_{a}}=(\Delta_{a}^{n}-\lambda_{a}^{n})$$

Then, we chose $\phi_{D}{}^{\prime }(\xi_{a})$ to have the form

(21) $$-\frac{\partial \phi_{D}(\xi_{a})}{\partial \xi_{a}}=\xi_{a}d$$

where *d* is a positive dimensionless material constant that is fitted in accordance with the maximum residual strain recorded during the experimental tests of the material samples. The integration of Equation (21) provides

(22) $$\phi_{D}(\xi_{a})=-\left(\frac{d}{2}\xi_{a}^{2}+d_{0}\right),$$

where *d*_0_ is an integration constant. Thus, from Equations (20) and (21) the following relationship is found

(23) $$\xi_{a}=\frac{1}{d}\left(\Delta_{a}^{n}-\lambda_{a}^{n}\right).$$

On the primary loading path, $\xi_{a}$ is inactive, while on the unloading path, $\xi_{a}$ has the value given by Equation (23). Substitution of Equation (23) into Equation (17) gives the following pseudo-elastic strain energy density expression per unit volume that accounts for residual strains on the unloading path:

(24) $$\hat{W}=\frac{1}{d}\sum_{a=1}^{3}\frac{1}{2}{\left(\Delta_{a}^{n}-\lambda_{a}^{n}\right)}^{2}+C_{0},$$

where *C*₀ is an integration constant that ensures that the residual strain energy density vanishes at the value of the corresponding unloading residual stretch deformation $\lambda_{res}$. Therefore, the strain energy function during unloading of an incompressible and hyperelastic material, in accordance with the pseudo-elastic theory, has the form:

(25) $${\bar{W}}_{T}(\lambda_{1},\lambda_{2},B_{i},\eta )=\eta W_{T}(\lambda_{1},\lambda_{2},B_{i})+\phi (\eta )+\frac{\eta_{1}}{d}\sum_{a=1}^{3}\frac{1}{2}{\left(\Delta_{a}^{n}-\lambda_{a}^{n}\right)}^{2}+C_{0}.$$

Notice from Equation (25) that the variable *η*₁ has the value of one on the loading path but decreases monotonically during unloading. Furthermore, and because of the expression that provides the dissipating energy due to residual strains given by Equation (24), during the loading of the material sample, the value of $\hat{W}$ is always zero. Therefore, the residual function *η*₁ can be assumed to have the same expression as that of *η* since this damage function is inactive during loading and decreases monotonically during unloading. Of course, other expressions can be used for *η*₁ so that 0 ≤ *η*₁ ≤ 1 is satisfied during unloading of the material sample. Thus, the energy dissipated during the loading–unloading cycle can be determined from:

(26) $$D=W_{T}-{\bar{W}}_{T}(\lambda_{1},\lambda_{2},B_{i},\eta ).$$

**Figure 1:**
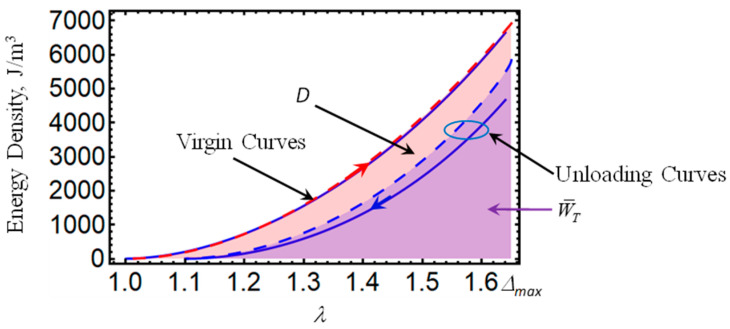
Schematic of the energy dissipated during one loading and unloading cycle of an isotropic magnetorheological polyurethane elastomer reinforced with 35 wt% of carbonyl iron microparticles. Notice that the value of *D* depends upon the point on the virgin curve at which the unloading process starts. Here *B* = 0, *μ*₀ = 13060 Pa, *N*₈ = 2.5, *f* = 0.076, *b* = 0.00275 m^3/2^J^1/2^, *A*₁ = 10000, *A*₂ = 30000, *d* = 0.0002 m²/N, and Δ*a* = 1.65.

The area enclosed by the loading and unloading curves shown in Figure 1 provides a schematic view of the energy dissipated during one cycle [17]. Moreover, the virgin material Cauchy-stress components are determined from:

(27) $$T_{0i}=-p+\lambda_{i}\frac{\partial W_{T}}{\partial \lambda_{i}}-\frac{\lambda_{i}}{2\mu }{\left(B_{1}^{Applied}\right)}^{2},\;\mathrm{where}\;i=1,2,3\;(\mathrm{no}\;\mathrm{sum})$$

in which Equation (7) has been considered. As usual, the undetermined pressure *p* can be eliminated by subtracting $T_{0i}$ from $T_{0j}$, this yields

(28) $$T_{0i}-T_{0j}=\left\{(1-f)ℵ+\frac{2f}{3}\left(A_{1}+\frac{2A_{2}}{3}(I_{1}-3)\right)\right\}(\lambda_{i}^{2}-\lambda_{j}^{2})-\frac{\lambda_{i}}{2\mu }{\left(B_{i}^{Applied}\right)}^{2}+\frac{\lambda_{j}}{2\mu }{\left(B_{j}^{Applied}\right)}^{2},$$

where i≠j=1,2,3, and

(29) $$ℵ=\frac{\mu_{0}}{3\lambda_{r}}\left[\beta +\frac{1}{N_{8}}\left(\frac{1}{\lambda_{r}}-\frac{1}{\beta (1-\lambda_{r}^{2}-2\lambda_{r}/\beta )}\right)\right].$$

Then, from the pseudo-elastic expression given by Equation (25), the stress-softened, permanent set, and magnetic effects can be predicted from

(30) $$\tau =T_{0}e^{-b\sqrt{W_{T\max}-W_{T}}},$$

where τ represents the Cauchy stress of the stress-softened material [9]. Of course, $\tau =T_{0},$ when and only when $W_{T}=W_{T\max}$. Thus, the stress-softened, residual strains and magnetic effects experienced by the magnetorheological elastomer during unloading is determined by the following equation

(31) $$\begin{array}{l} \tau_{0i}-\tau_{0j}=[\{(1-f)ℵ+\frac{2f}{3}\left(A_{1}+\frac{2A_{2}}{3}(I_{1}-3)\right)\}(\lambda_{i}^{2}-\lambda_{j}^{2})-\frac{\lambda_{i}}{2\mu }{\left(B_{i}^{Applied}\right)}^{2}+\\ \frac{\lambda_{j}}{2\mu }{\left(B_{j}^{Applied}\right)}^{2}+\frac{n}{d}(\lambda_{i}^{n}(\lambda_{i}^{n}-\Delta_{i}^{n})-\lambda_{j}^{n}(\lambda_{j}^{n}-\Delta_{j}^{n}))]e^{-b\sqrt{W_{T\max}-W_{T}}}, \end{array}$$

Here, *n* is assumed to have the value of 1/2, *d* is a positive residual strain material constant, $\Delta_{i},\;i=1,2,3$ (no sum) are the principal maximum stretch values at the point on the loading virgin curve for which unloading of the material sample begins. Of course, the uniaxial engineering stress tensor can be computed by using the relationship $\sigma =TF^{-1}$. We shall next investigate the accuracy of the derived expressions for energy–stretch and stress–stretch to predict experimental data in which residual strains and magnetic effects are considered.

## 4. Comparison with Experimental Data

We next examine the accuracy of the introduced model to predict the Mullins and residual strain effects in the produced isotropic and anisotropic magnetorheological material samples reinforced with 20, 35, 50, 65, and 80 wt% of carbonyl iron microparticles when subjected to cyclic loading–unloading uniaxial extension tests, with and without the action of a magnetic flux density.

### Numerical Results

First, the strain energy density curves of the material samples were computed by using uniaxial extension experimental data and Equations (7) and (25). The first, second, third, and fourth columns of Table 1 and Table 2 summarize the different parameter values considered for the fabrication and experimental tests of the material samples. The remaining columns of Table 1 and Table 2 show the estimated material constant values obtained by best fitting of the experimental data. Table 3 and Table 4 show the material constant for each test cycle. In addition, the maximum and residual stretch values collected during experimental tests are listed for each loading–unloading cycle.

**Figure 2:**
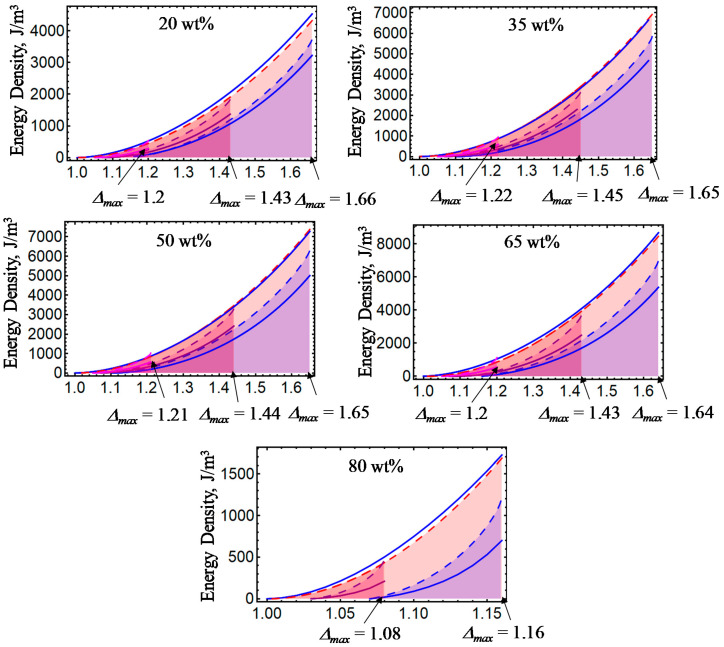
Energy dissipation curves for isotropic magnetorheological material samples subjected to loading and unloading cycles with *B* = 0 mT. Notice that for increasing wt% of CIPs, the dissipation area broadens, which is an indication of the damage experienced in the material during cyclic loading.

**Figure 3:**
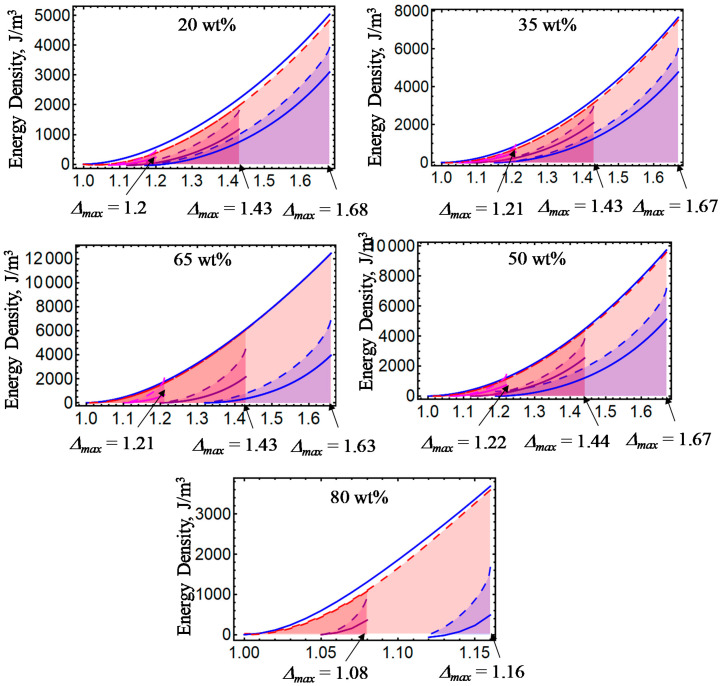
Energy dissipation energy curves for isotropic magnetorheological material samples subjected to loading and unloading cycles with *B* = 52.2 mT.

**Figure 4:**
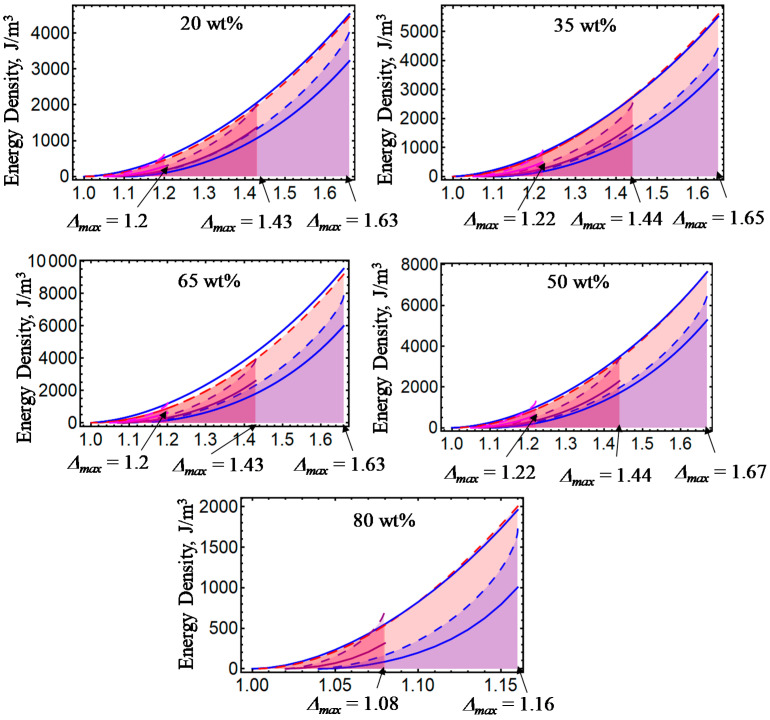
Energy dissipation energy curves for anisotropic magnetorheological material samples subjected to loading and unloading cycles with *B* = 0 mT.

**Figure 5:**
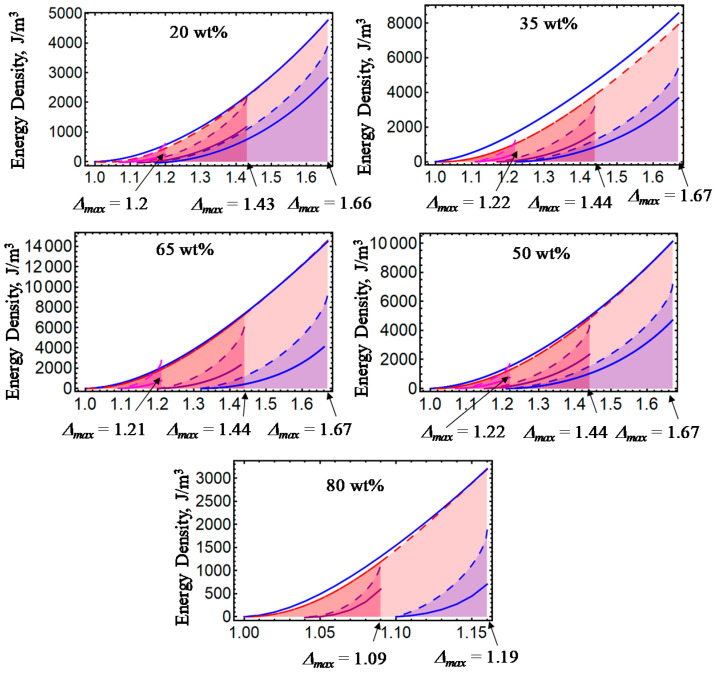
Energy dissipation energy curves for anisotropic magnetorheological material samples subjected to loading and unloading cycles with *B* = 52.2 mT.

Figure 1, Figure 2, Figure 3, Figure 4 and Figure 5 illustrate the energy dissipation as a function of the amount of stretch to which the material samples were subjected during loading–unloading cycles. Here, the colored solid lines define the strain energy density obtained from experimental data, while the dashed colored lines are theoretical predictions computed from Equations (7) and (25). Notice that *D* is sensitive to the different wt% of CIPs concentrations, to the material properties (isotropic or anisotropic), to the maximum amount of elongation at which the samples are subjected, to the resulting residual strains, and to the magnetic flux density. The discrepancies observed between experimental data and theoretical predictions are mainly due to the assumption that viscoelastic effects are negligible, and that pseudo-elastic theory can be used to predict the energy dissipated during unloading of the material samples. However, in spite of having ignored viscoelastic effects during the derivation of Equations (12) and (24), the theoretical energy curves shown in Figure 1, Figure 2, Figure 3, Figure 4 and Figure 5 describe the materials’ qualitative and quantitative behaviors well.

Since the energy dissipated for each loading–unloading cycle is expected to increase with an increase in the elastic energy $W_{T}$, the dissipation factor

(32) $$E=\frac{D}{W_{T\max}}$$

is introduced in an attempt to quantify the differences among the tested material samples. This dissipation factor differs from that proposed by Mai et al. in [17] since in our definition, we compute the ratio of energy dissipation to the maximum strain energy density at which unloading starts, while in the definition introduced in [17], the dissipation ratio is defined as the ratio of the energy dissipation to the strain energy density of the loading curve.

**Figure 6:**
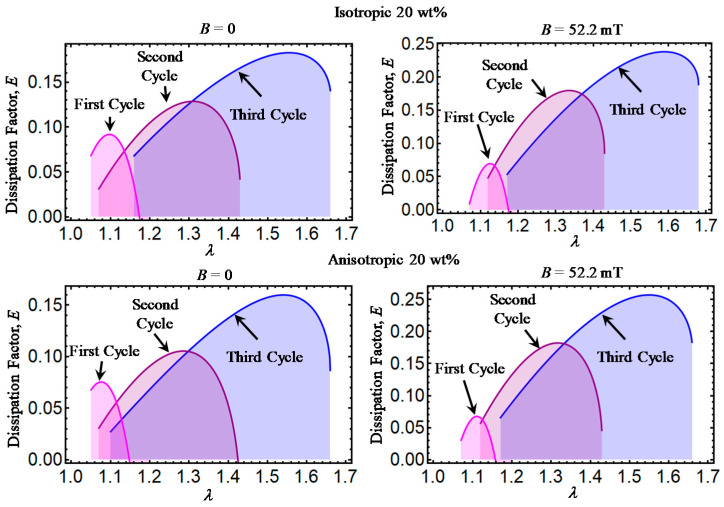
Dissipation factor curves for magnetorheological material samples made with a concentration of 20 wt% of CIPs. A high value of *E* indicates that the energy loss per loading–unloading cycle is small. The higher the *E,* the lower the stress-softening and residual strain effects related to the molecular material damage. Also, notice that the area under the curve indicates the material capacity to dissipate energy per loading–unloading cycle.

**Figure 7:**
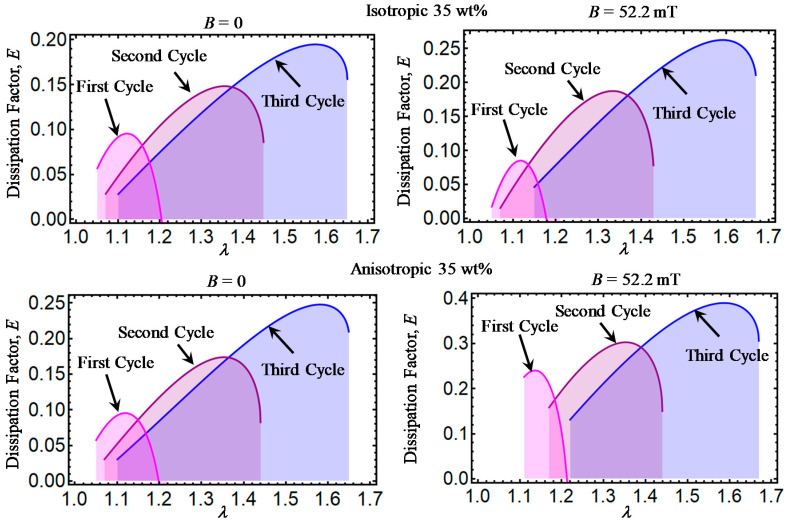
Dissipation factor curves for magnetorheological material samples made with a concentration of 35 wt% of CIPs.

**Figure 8:**
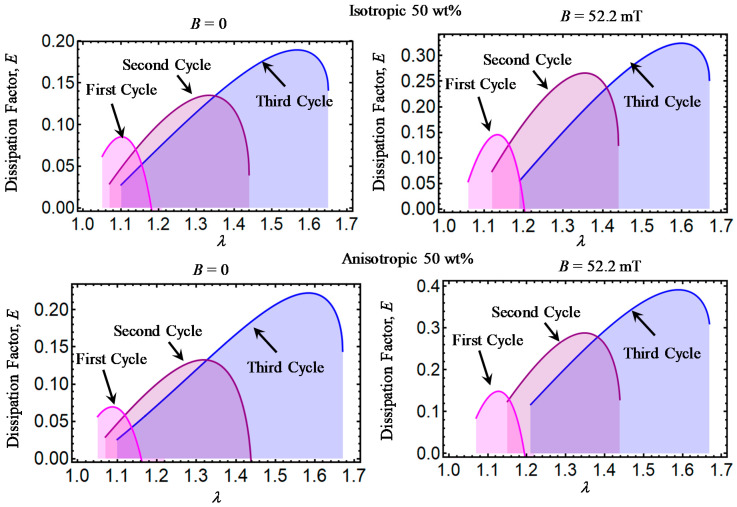
Dissipation factor curves for magnetorheological material samples made with a concentration of 50 wt% of CIPs.

**Figure 9:**
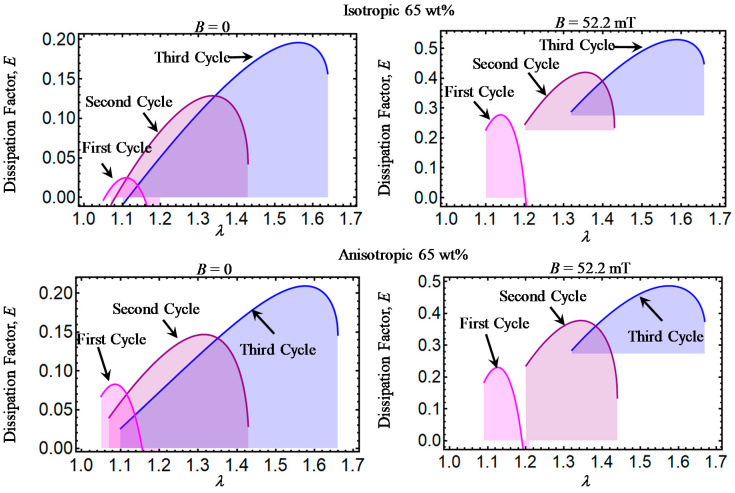
Dissipation factor curves for magnetorheological material samples made with a concentration of 65 wt% of CIPs.

**Figure 10:**
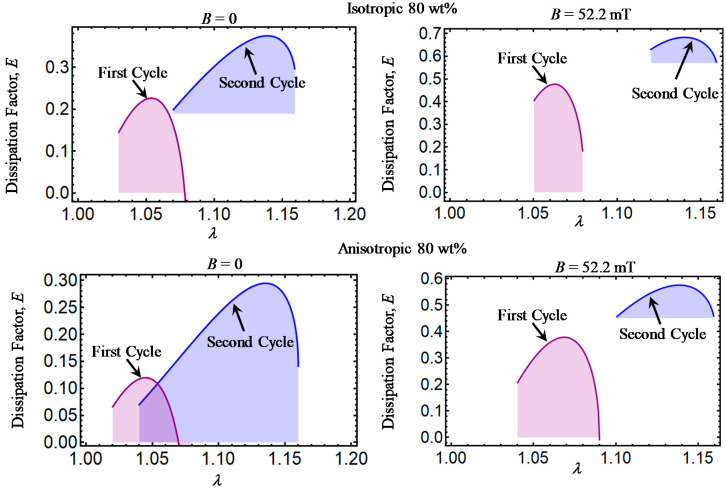
Dissipation factor curves for magnetorheological material samples made with a concentration of 80 wt% of CIPs.

Theoretical predictions of the dissipation factor *E* computed from Equation (32) are illustrated in Figure 6, Figure 7, Figure 8, Figure 9 and Figure 10. Here, *E* has been plotted versus amount of stretch for the various concentrations of CIPs used to develop the material samples. The area under each dissipation factor curve indicates the material capacity to dissipate energy per loading–unloading cycle. In other words, the higher the area, the higher the material damage that induces inelastic phenomena, such as stress-softening and residual strain effects.

Also, a low value of E indicates that the energy loss per loading-unloading cycle is small. The value of *E* could also provide information about the debonding process, since a decrease in the material shear modulus is evident after the application of the first loading–unloading cycle.

When the material samples are further subjected to loading–unloading cycles, the Mullins effect as well as the residual strains increase with a noticeable decrease in the material shear modulus, especially for those composite samples reinforced with CIPs concentrations of 35, 50, 65, and 80 wt%. It is also observed that the material shear modulus becomes sensitive not only to the wt% of CIPs added into the elastomer material matrix, but also due to the application of the magnetic flux intensity that induces attractive forces between CIPs and to the strong bonds between these and the elastomer matrix.

However, for the material samples with CIPs concentration of 20 wt%, it appears that the material shear modulus is not sensitive to the application of a magnetic flux density, as confirmed in [2] for small wt% concentrations of CIPs mixed with the elastomer matrix.

**Figure 11:**
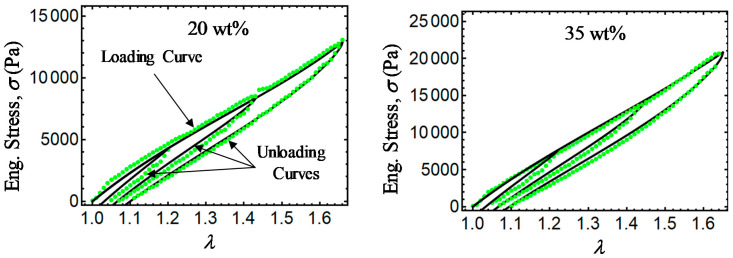

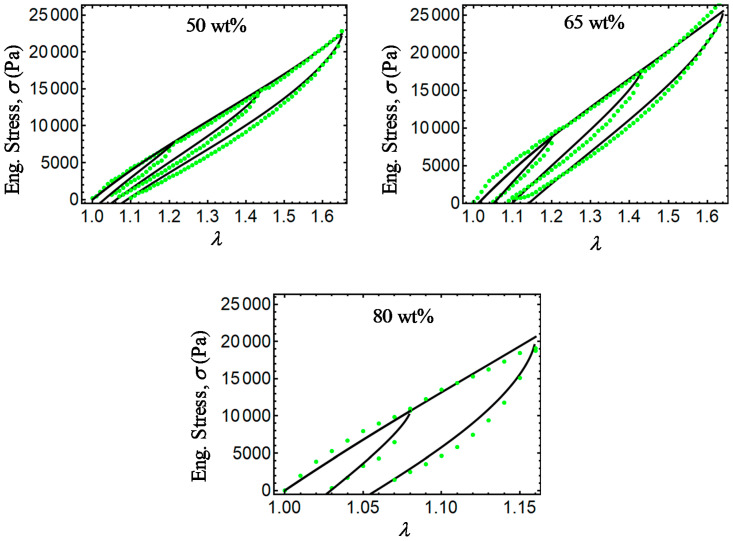
Engineering stress–stretch curves for isotropic magnetorheological material samples with *B* = 0 mT. Here, the green dots describe experimental data, while the black solid lines are theoretical predictions obtained from Equations (28) and (31).

**Figure 12:**
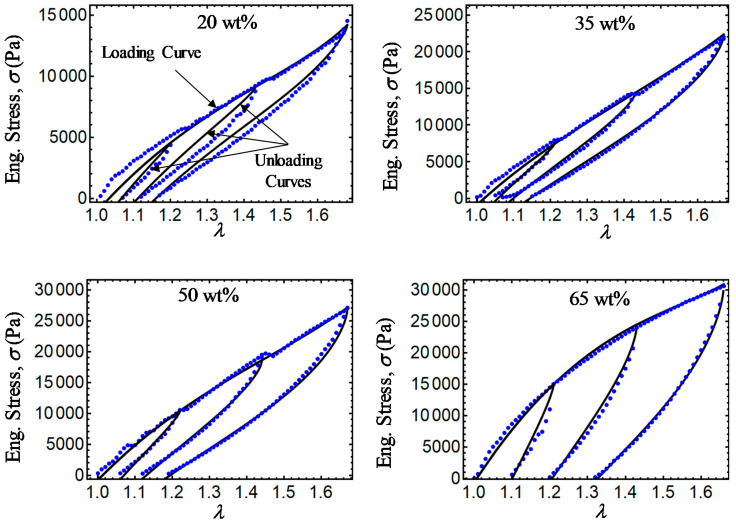

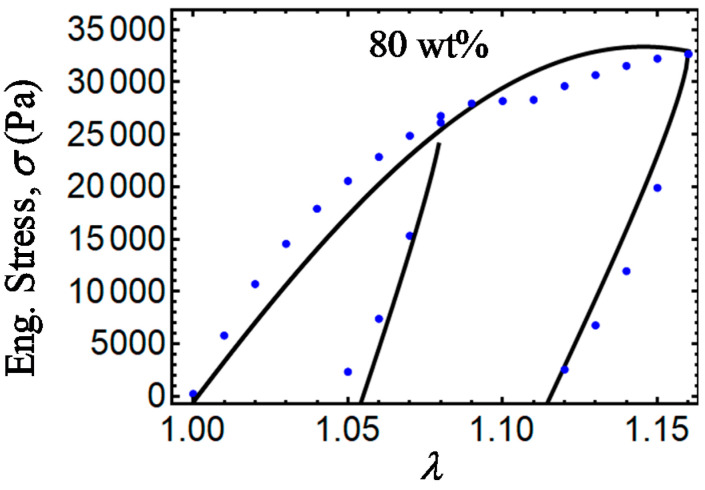
Engineering stress–stretch curves for isotropic magnetorheological material samples with *B* = 52.2 mT. Here, the blue dots describe experimental data while the black solid lines are theoretical predictions obtained from Equations (28) and (31).

**Figure 13:**
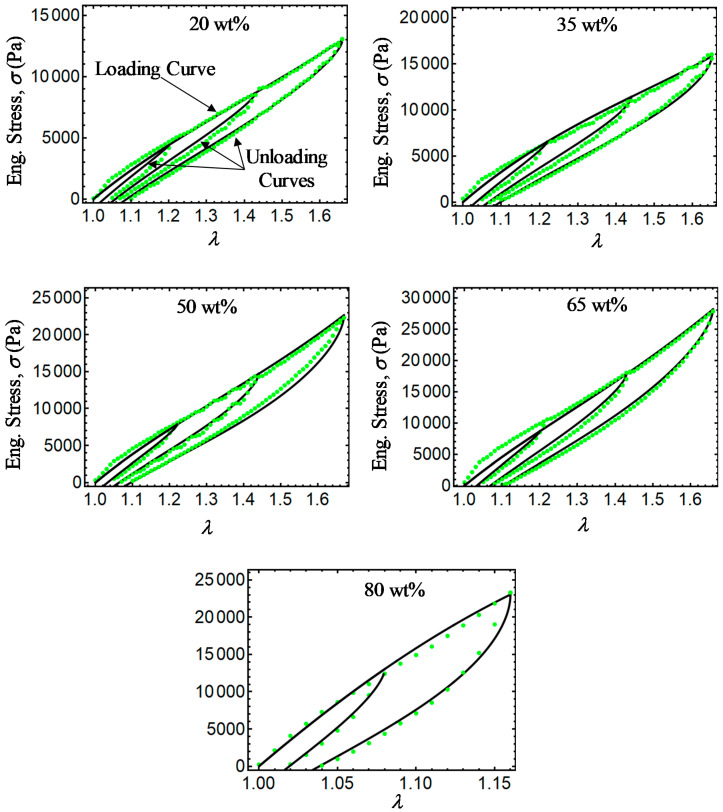
Engineering stress–stretch curves for anisotropic magnetorheological material samples with *B* = 0 mT. Here, the green dots describe experimental data while the black solid lines are theoretical predictions obtained from Equations (28) and (31).

**Figure 14:**
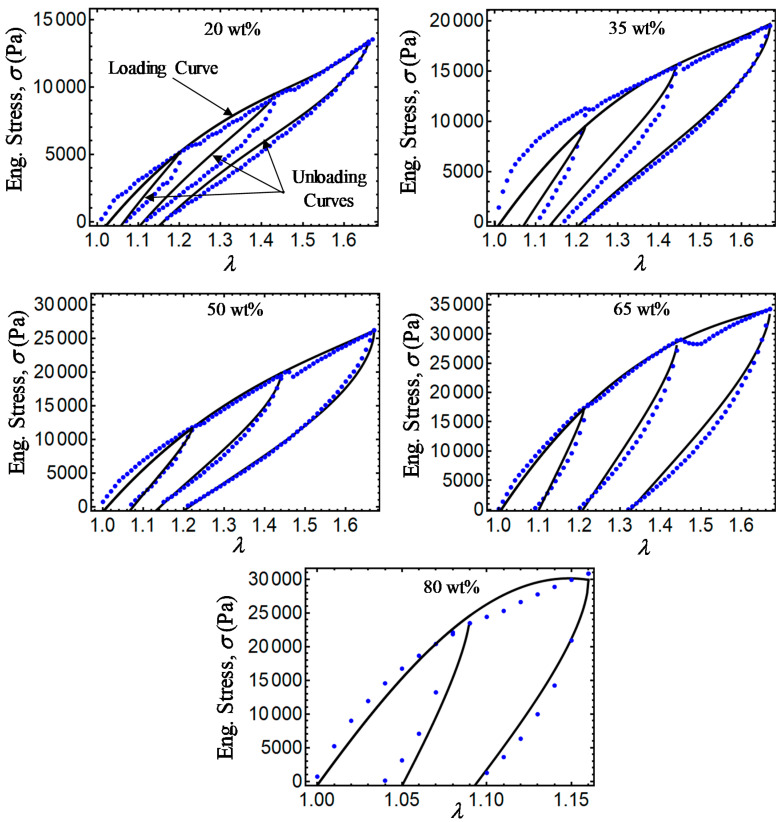
Engineering stress–stretch curves for anisotropic magnetorheological material samples with *B* = 52.2 mT. Here, the blue dots describe experimental data, while the black solid lines are theoretical predictions obtained from Equations (28) and (31).

Finally, Figure 11, Figure 12, Figure 13 and Figure 14 show a comparison of experimental data with simulation results obtained from Equations (28) and (31) and the relationship $\sigma =T_{0}F^{-1}$. Note that there is a slight difference between experimental data and the energy-based Equations (28) and (31) in predicting stress-softening and residual strain effects. From Figure 11, Figure 12, Figure 13 and Figure 14, it can be concluded that the addition of CIPs with a predefined orientation of the microparticles embedded into the polymer matrix and the application of a magnetic flux density in the loading–unloading direction could enhance the material shear modulus as well as its capacity to dissipate energy.

## 5. Material Preparation

In what follows, some aspects regarding the preparation and development of the magnetorheological polyurethane material samples are discussed.

### 5.1. Materials

Materials used to manufacture the polyurethane magnetorheological elastomers were two urethane rubbers (URs) Vytaflex@10 with a mixed viscosity of 3100 cps and a shore hardness of 10 A, both purchased from Smooth-On Inc. (Macungie. PA, USA), as well as the phthalate free softening agent SO Flex II Vitaflex part A consisting of toluene diisocyanate and diisononylphthalate and the vytaflex part B consisting of a mix of ether polyol and plasticizer and diethyltoluenediamine. The spherical carbonyl iron particles, with an average size of 2.25 µm, were purchased from Sigma-Aldrich (Toluca, Mexico).

### 5.2. Magnetorheological Material Preparation

To prepare the elastomer, one-to-one by volume mix ratios of Part A and B of the URs were mixed. Several composite material samples were produced by considering CIPs concentrations of 20, 35, 50, 65, and 80 wt%. These CIPs concentrations were mixed with the SO Flex II until homogenous mixtures formed. Part B of the UR was mixed before it was poured into the CIPs mixture. Then, part A of the UR was mixed and added to the last mixture, and the whole solution was homogeneously mixed for a few minutes before it was poured, at room temperature, into different molds. All composite material samples were mixed at room temperature and subjected to vacuum conditions during the curing process to avoid porosity. Anisotropic magnetorheological elastomers were produced by applying, during the curing process, a magnetic flux density of 52.2 mT for 30 min.

**Figure 15:**
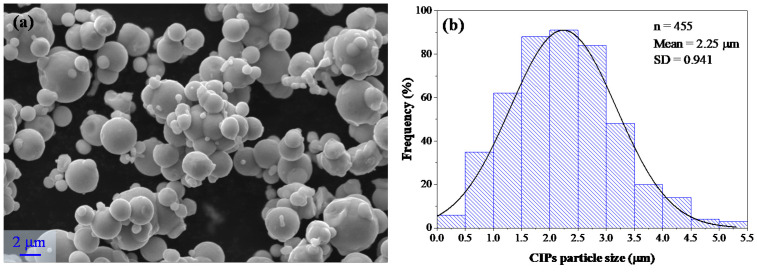
(**a**) Morphology of carbonyl iron particles (powder), and (**b**) diameter particle size distribution computed by using Digimizer 4.6.1 software (MedCalc Ltd.).

Figure 15a depicts the morphology of the magnetic particle powder used to manufacture the magnetorheological materials. As it can be seen, the CIPs have a spherical morphology of micron-size particles. Figure 15b shows the computed histogram of the size distribution of the CIPs. For estimating the average size of CIPs, the diameter of 455 particles were measured through the Digimizer 4.6.1 software (MedCalc Ltd., Ostend, Belgium). It was found that the magnetic particles have a diameter range of 0.25 to 5.25 µm, and an average diameter of 2.25 µm, as shown in Figure 15b.

**Figure 16:**
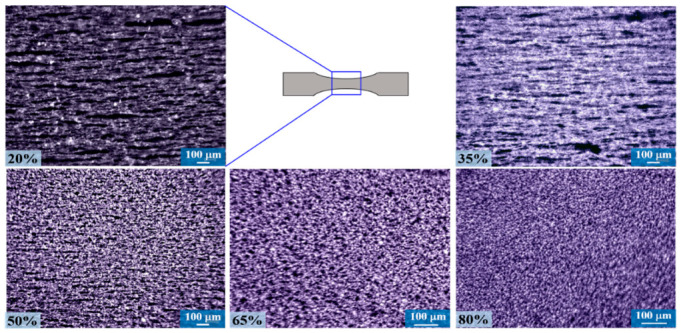
Images that show the alignment for an anisotropic sample of the CIPs inside the polyurethane (PU) matrix when subjected to a magnetic flux density of 52.2 mT.

The magnetorheological samples were analyzed by optical microscopy to observe the distribution of CIPs in the PU matrix. Figure 16 shows the images of anisotropic samples for each amount of CIPs added to the polymeric matrix. Notice from Figure 16 that the CIPs are aligned parallel to the orientation of the magnetic field applied during the curing process. This alignment is evident for the sample that contains 20 wt% of CIPs. However, as the wt% of CIPs increases, microparticle saturation hinders the alignment because of the decreasing of the polymeric matrix mass content [11].

### 5.3. Uniaxial Extension Tests

**Figure 17:**
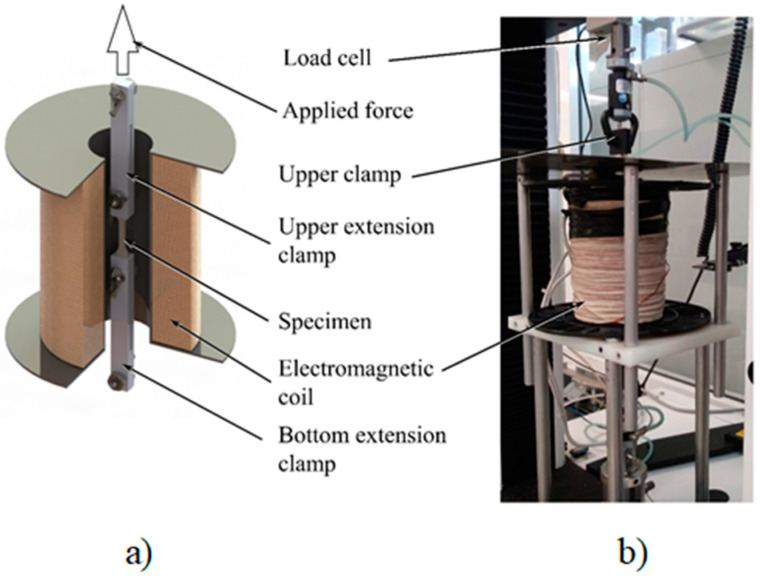
(**a**) Schematic representation of the solenoid, extension clamps, and specimen. (**b**) Uniaxial tensile machine coupled with a solenoid coil to perform the cyclic tensile test under a magnetic field.

Once the magnetorheological mixtures were cured, the different material samples were subjected to cyclic uniaxial extension tests in an Instron 3365 universal testing machine, with bounding box dimensions of 2.1 × 0.76 × 0.71 m. In order to have better force measurement, a static load cell of 5 N (Model: 2530-5N) was used. The specimen’s dumbbell-shaped form was defined under the norm ISO37-2011. The material samples were located inside a solenoid coil so that a magnetic field was oriented towards the direction of the tensile load applied by the testing machine, as shown in Figure 17. The samples were stretched to a maximum elongation of 20 mm, with a crosshead rate of 200 mm/min, to have a uniform magnetic flux density acting on the material samples during loading–unloading cycles. All the samples were kept inside the solenoid coil during the magnetic flux density application. The samples were stretched, *λ*, for three loading–unloading cycles with maximum elongation values of 7, 14, and 20 mm, whereby *λ =* 1 + *L_f_*/*L_i_*. Here, *L_i_* and *L_f_* are the initial and the final sample lengths, respectively.

**Figure 18:**
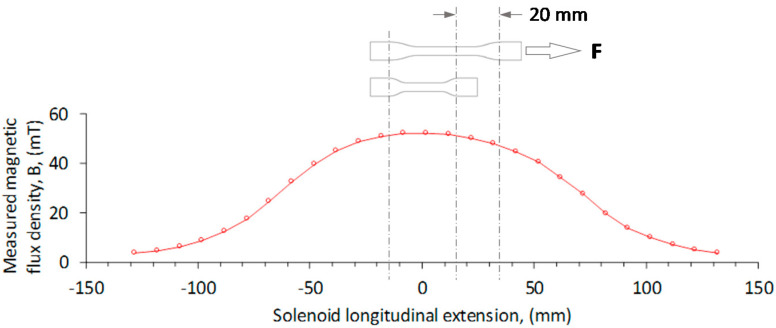
Magnetic flux intensity applied to the specimen inside the solenoid during cyclic loading–unloading tensile tests. F is the tensile force applied during the test.

**Figure 19:**
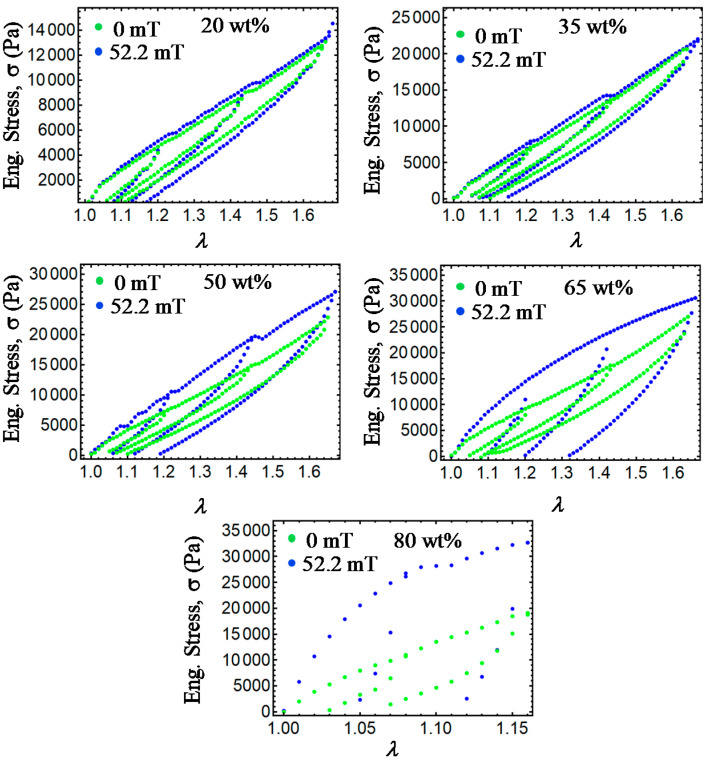
Collected experimental data for isotropic magnetorheological material samples subjected to loading and unloading cycles with and without the action of a magnetic flux density. Notice that for increasing wt% of CIPs, the material shear moduli, the engineering stress, the dissipation of energy, and residual strains tend to increase.

**Figure 20:**
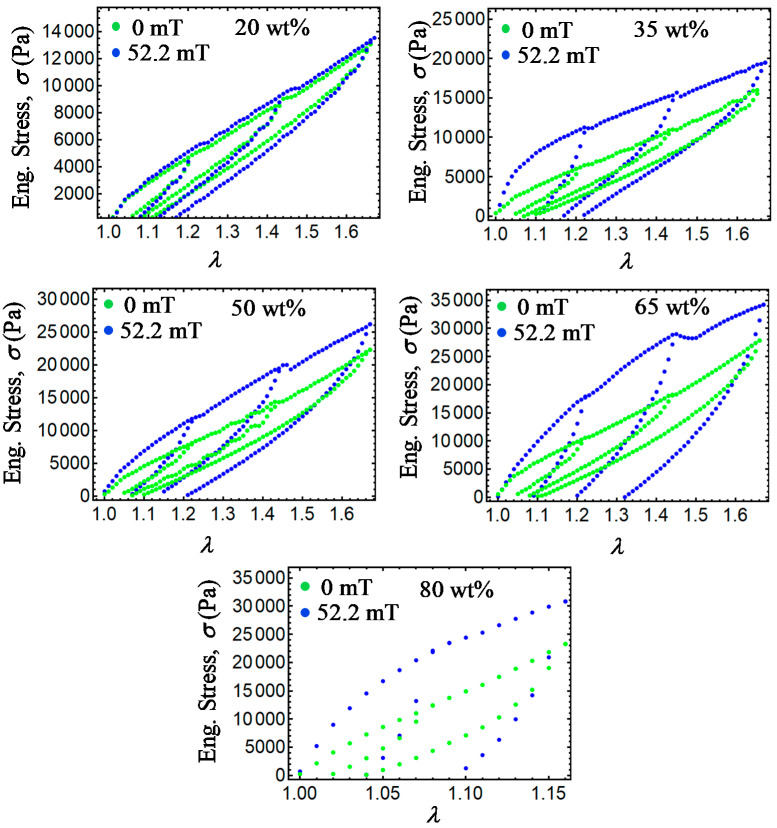
Collected experimental data for anisotropic magnetorheological material samples subjected to loading and unloading cycles with and without the action of a magnetic flux density. Notice that for increasing wt% of CIPs, the material shear moduli, the engineering stress, the dissipation of energy, and residual strains tend to increase.

The maximum stretch elongation values were increased in general, from Δ*a* = 1.22, 1.44, to 1.64. In all cases, the specimens were subjected to the magnetic flux density of 52.2 mT exerted by the solenoid shown in Figure 18, with a maximum flux variation value of 4%, as recorded by the longitudinal probe of a Gaussmeter BELL-5170 apparatus used to measure the magnetic flux density along the solenoid longitudinal axis. Details of the solenoid coil used during experimental tests are described in [11]. Experimental tests for the loading–unloading cycles were performed an average of five times in order to confirm reproducibility. Figure 19 and Figure 20 illustrate the isotropic and anisotropic material samples’ behavior with and without the action of a magnetic flux density during the loading–unloading uniaxial extension cycles. In both Figure 19 and Figure 20, permanent set and stress-softened effects occur. When the samples are subjected to a magnetic flux density, the material tensile stress increases for higher concentrations of CIPs. In fact, an increase of the material stress of about 785% and 744% were achieved when comparing the isotropic and anisotropic samples made with 80 wt% with respect to those made with 20 wt% of CIPs at the amount of stretch *λ* = 1.16.

## 6. Conclusions

Stress-softening, residual strains, and energy dissipated in loading–unloading cycles were evaluated by considering the wt% of CIPs concentration added into the polyurethane elastomer matrix, the isotropic and anisotropic material properties, and the impact of an applied constant magnetic flux density. We found that when the wt% of CIPs increases, the energy dissipated increases, especially for higher concentrations of CIPs. Furthermore, the Mullins and residual strain effects increase for those material samples for which the CIPs embedded along the application of the uniaxial force and the magnetic flux density. In other words, it was found that for the anisotropic samples reinforced with CIPs concentrations higher than 20 wt% when subjected to uniaxial extension cyclic loads, exhibit higher shear modulus than the isotropic ones, which becomes evident for those material samples subjected to a 52.2 mT of magnetic flux density and reinforced with 35, 50, 65, and 80 wt% of CIPs. Moreover, the energy dissipated during the loading–unloading cycles increases because of the wt% of CIPs added into the elastomer matrix when the samples are under the action of a magnetic flux density, as confirmed by the maximum value achieved by the dissipation factor curves. This material behavior is an indication that the Mullins and residual strain effects become sensitive to the wt% of CIPs added into the elastomer matrix and to the application of a magnetic flux density. Therefore, it is concluded that in those material samples made from CIPs with a predefined orientation when subjected to cyclic loads and to the action of a magnetic flux density, their shear modulus and their capacity to dissipate energy increases. These conclusions are important since knowing in advance the degree of damage experienced by these materials and the impact of that damage on their physical and mechanical properties when subjected to external cyclic loads and magnetic fields will allow for the enhancement of the applicability of polyurethanes. Polyurethanes are already widely used in biomedical, automotive, construction, textiles, and other industrial applications because of their physical properties, such as resistance to corrosion, durability, strength, elongation, toughness, shrinkage, and expansion, among others, that makes these elastomers suitable for devices under the action of a magnetic field.

## Figures and Tables

**Table 1:** Estimated material constant values of *μ*₀, *μ*_r_, N₈, *b*, *A*₁, *A*₂, *d*, and *c*₁ obtained by fitting experimental data with B=0 mT.

wt%	Particle Arragmt	*f*	Particle Arragmt	$\mu_{0}$ N/m^2^	$\mu_{r}$	$N_{8}$	b (m^3^/J)^1/2^	$A_{1}$	$A_{2}$	d m^2^/N	$c_{1}$ J/m^3^
20	ISO	0.037	ISO	12,989	0	2.1	0.0025	−210,000	−80,000	0.0031	3595.7199
20	ANI	0.037	ANI	10,285	0	7.5	0.0035	−65,000	70,000	0.00035	3756.6586
35	ISO	0.076	ISO	13,060	0	2.5	0.00275	10,000	30,000	0.0002	3798.4371
35	ANI	0.076	ANI	9889	0	2	0.005	40,000	−60,000	0.00022	2537.1349
50	ISO	0.132	ISO	16,637	0	2.25	0.003	−15,000	10,000	0.0002	4325.5832
50	ANI	0.132	ANI	16,275	0	2.5	0.005	−15,000	20,000	0.0002	4446.6251
65	ISO	0.221	ISO	20,763	0	4.5	0.003	10,000	15,000	0.0001	5198.5202
65	ANI	0.221	ANI	19,427	0	4.5	0.0035	5000	45,000	0.00015	5472.2308
80	ISO	0.379	ISO	66,096	0	1.2	0.0125	20,000	−100,000	0.000018	1177.5485
80	ANI	0.379	ANI	6374	0	5.3	0.015	220000	−380000	0.000026	1459.0584

**Table 2:** Estimated material constant values of *μ*₀, *μ*_r_, N₈, *b*, *A*₁, *A*₂, *d*, and *c*₁ obtained by fitting experimental data with B=52.2 mT.

wt%	Particle Arragmt	*f*	$\mu_{0}$ N/m^2^	$\mu_{r}$	$N_{8}$	b (m^3^/J)^1/2^	$A_{1}$	$A_{2}$	d m^2^/N	$c_{1}$ J/m^3^
20	ISO	0.037	13,154	1.01	1.875	0.0025	−100,000	−250,000	0.00018	2234.0156
20	ANI	0.037	16,720	1.01	1.8	0.0035	−190,000	−480,000	0.00015	2961.9939
35	ISO	0.076	14,631	1.23	2.1	0.004	18,000	−30,000	0.00013	2987.8284
35	ANI	0.076	20,607	1.23	1.9	0.0053	40,000	−275,000	0.00006	3429.1346
50	ISO	0.132	19,845	1.47	1.9	0.005	20,000	−80,000	0.000063	3830.2696
50	ANI	0.132	21,695	1.47	2.1	0.006	30,000	−120,000	0.000049	4661.5804
65	ISO	0.221	32,906	1.7	1.845	0.006	20,000	−160,000	0.000019	5978.7898
65	ANI	0.221	48,534	1.7	2.3	0.0045	−40,000	−160,000	0.000017	10806
80	ISO	0.379	135,132	1.93	1.225	0.0075	170,000	−4,700,000	0.0000019	3518.4793
80	ANI	0.379	140,833	1.93	1.2	0.0125	100,000	−4,200,000	0.0000035	1936.4788

**Table 3:** Estimated material constant values of *C*₀ obtained by fitting experimental data. The listed values of $\Delta_{a}$ and λ*_res_* were recorded for each loading–unloading cyclic experimental test with B=0 mT.

wt%	$\Delta_{a}$	$\lambda_{res}$	$C_{0}$ J/m^3^	wt%	$\Delta_{a}$	$\lambda_{res}$	$C_{0}$ J/m^3^
Isotropic Samples				Anisotropic Samples			
20	1.66	1.16	4171.9537	20	1.66	1.1	4143.3076
20	1.43	1.07	3652.761	20	1.43	1.07	3668.8424
20	1.2	1.05	3507.3879	20	1.2	1.05	3585.467
35	1.65	1.1	4879.1067	35	1.65	1.1	3662.2473
35	1.45	1.07	4054.7228	35	1.44	1.07	2764.5743
35	1.22	1.05	3691.0305	35	1.22	1.05	2400.5812
50	1.65	1.1	5369.6776	50	1.67	1.1	5550.4437
50	1.44	1.07	4464.056	50	1.44	1.07	4325.2313
50	1.21	1.05	4149.4913	50	1.22	1.05	4056.1777
65	1.64	1.1	6612.4138	65	1.66	1.1	6608.6042
65	1.43	1.07	5406.081	65	1.43	1.07	5237.3154
65	1.2	1.05	4933.1887	65	1.2	1.05	4959.4039
80	1.16	1.07	1793.3486	80	1.16	1.04	1793.3486
80	1.08	1.03	1349.649	80	1.08	1.02	1349.649

**Table 4:** Estimated material constant values of *C*_0_ obtained by fitting experimental data. The listed values of $\Delta_{a}$ and λ*_res_* were recorded for each loading–unloading cyclic experimental test with B=52.2 mT.

wt%	$\Delta_{a}$	$\lambda_{res}$	$C_{0}$ J/m^3^	wt%	$\Delta_{a}$	$\lambda_{res}$	$C_{0}$ J/m^3^
Isotropic Samples				Anisotropic Samples			
20	1.68	1.17	3146.7057	20	1.66	1.17	3773.2832
20	1.43	1.12	2402.4987	20	1.43	1.12	3017.1163
20	1.2	1.07	2147.5641	20	1.2	1.07	2774.2649
35	1.67	1.15	4490.2521	35	1.67	1.22	5849.0836
35	1.43	1.07	3173.61	35	1.44	1.17	4008.5378
35	1.21	1.05	2768.8979	35	1.22	1.11	3235.8117
50	1.67	1.19	6242.5993	50	1.67	1.21	7623.528
50	1.44	1.12	4375.9	50	1.44	1.15	5141.7377
50	1.22	1.06	3533.2046	50	1.22	1.07	4151.7441
65	1.66	1.32	11338.1253	65	1.67	1.32	15975.7794
65	1.43	1.2	7412.05	65	1.44	1.2	11790.501
65	1.21	1.1	5567.3315	65	1.21	1.09	9928.1565
80	1.16	1.12	5419.9761	80	1.16	1.1	3773.2833
80	1.08	1.05	3590.1389	80	1.09	1.04	3017.1163
